# Scanning the active center of formolase to identify key residues for enhanced C1 to C3 bioconversion

**DOI:** 10.1186/s40643-024-00767-3

**Published:** 2024-05-12

**Authors:** Guimin Cheng, Hongbing Sun, Qian Wang, Jinxing Yang, Jing Qiao, Cheng Zhong, Tao Cai, Yu Wang

**Affiliations:** 1 College of Biotechnology, Tianjin University of Science and Technology, Tianjin, 300222 People’s Republic of China; 2 Haihe Laboratory of Synthetic Biology, Tianjin, 300308 China; 3 Key Laboratory of Engineering Biology for Low-carbon Manufacturing, Tianjin Institute of Industrial Biotechnology, Chinese Academy of Sciences, Tianjin, 300308 People’s Republic of China; 4 School of Biology and Biological Engineering, South China University of Technology, Guangzhou, 510006 China; 5 National Center of Technology Innovation for Synthetic Biology, Tianjin, 300308 China

**Keywords:** Formolase, Dihydroxyacetone, C1 bioconversion, Carbon fixation, Synthetic methylotrophy

## Abstract

**Background:**

Formolase (FLS) is a computationally designed enzyme that catalyzes the carboligation of two or three C1 formaldehyde molecules into C2 glycolaldehyde or C3 dihydroxyacetone (DHA). FLS lays the foundation for several artificial carbon fixation and valorization pathways, such as the artificial starch anabolic pathway. However, the application of FLS is limited by its low catalytic activity and product promiscuity.

**Findings:**

FLS, designed and engineered based on benzoylformate decarboxylase from *Pseudomonas putida*, was selected as a candidate for modification. To evaluate its catalytic activity, 25 residues located within an 8 Å distance from the active center were screened using single-point saturation mutagenesis. A screening approach based on the color reaction of the DHA product was applied to identify the desired FLS variants. After screening approximately 5,000 variants (approximately 200 transformants per site), several amino acid sites that were not identified by directed evolution were found to improve DHA formation. The serine-to-phenylalanine substitution at position 236 improved the activity towards DHA formation by 7.6-fold. Molecular dynamics simulations suggested that the mutation increased local hydrophobicity at the active site, predisposing the cofactor-C2 intermediate to nucleophilic attack by the third formaldehyde molecule for subsequent DHA generation.

**Conclusions:**

This study provides improved FLS variants and valuable information into the influence of residues adjacent to the active center affecting catalytic efficiency, which can guide the rational engineering or directed evolution of FLS to optimize its performance in artificial carbon fixation and valorization.

**Supplementary Information:**

The online version contains supplementary material available at 10.1186/s40643-024-00767-3.

## Background

CO_2_ is a fundamental carbon resource for the synthesis of various foods, chemicals, and fuels (Liu et al. 2023; Wang et al. 2015). Engineering biological systems capable of converting C1 compounds into multi-carbon molecules is an important approach for the fixation and valorization of CO_2_ (Siegel et al. 2015). In addition to the direct sequestration of CO_2_ by photosynthesis in plants and cyanobacteria (Tan et al. 2022), integrated chemical-biological routes for artificial food and material synthesis from CO_2_ have been established, which consist of the chemical reduction of CO_2_ into organic C1 or C2 compounds, such as methanol or acetate, followed by the biosynthesis of multi-carbon molecules facilitated by enzymes or cells (Cai et al. 2021; Hann et al. 2022; Yang et al. 2023; Zhang et al. 2023; Zheng et al. 2022).

**Fig. 1:**
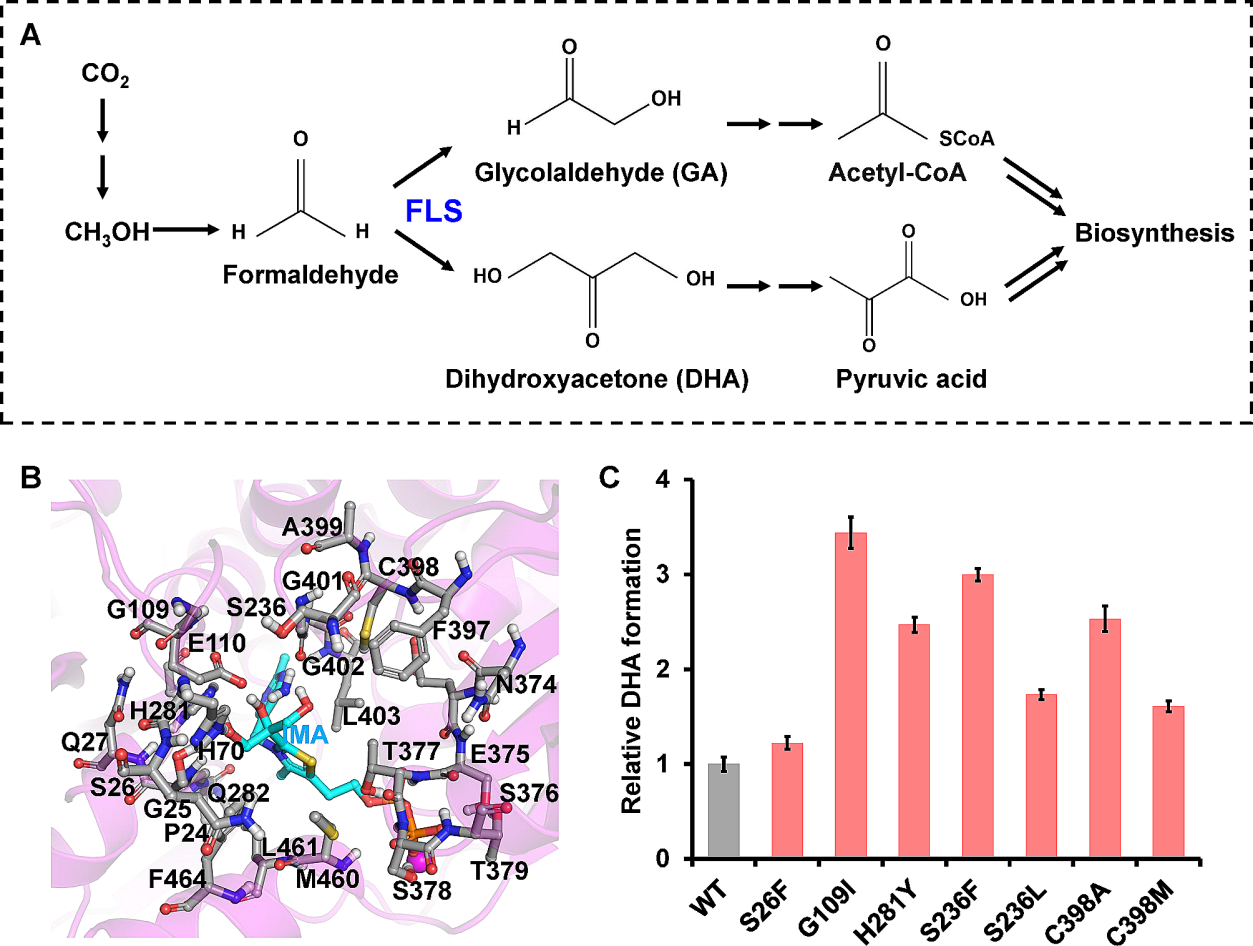
Engineering of FLS for artificial C1 bioconversion. **A** Schematic illustration of FLS-mediated artificial C1 bioconversion for biosynthesis. **B** Selected residues for single-point saturation mutation. The 25 residues within an 8 Å distance from the product intermediate analogue (IMA) are represented in grey, and thiamine diphosphate (TPP) is represented in cyan. **C** Seven amino acid substitutions beneficial for DHA formation were identified by screening of the FLS variants with single-point mutations. Values and error bars reflect the mean ± s.d. of three biological replicates

Enzymes that catalyze carboligation reactions are considered key catalysts for artificial CO_2_ bioconversion. Formolase (FLS) is a computationally designed enzyme based on benzaldehyde lyase from *Pseudomonas fluorescens* and benzoylformate decarboxylase (BFD) from *P. putida*. FLS catalyzes the formation of one C2 glycolaldehyde (GA) or C3 dihydroxyacetone (DHA) from two or three C1 formaldehyde molecules, respectively (Lu et al. 2019; Siegel et al. 2015). The conversion of C1 to C3 by FLS facilitates the construction of artificial anabolic pathways for the synthesis of starch, sugars, and lactate as well as the engineering of synthetic methylotrophs (Fig. 1A) (Cai et al. 2021; Li et al. 2020; Lu et al. 2019; Wang et al. 2017; Wu and Bornscheuer, 2022; Yang et al. 2023; Yang et al. 2017). However, the catalytic activity of FLS is relatively low; making it a major limiting factor in the artificial C1 bioconversion process. In addition, FLS exhibits a low preference for the C3 product DHA, which further limits its application (Cai et al. 2021; Qiao et al. 2023).

To address this issue, a directed evolution of the FLS has been conducted. A growth-couple screening method was developed based on the principle of formaldehyde detoxification, through which an FLS variant with a 2.3-fold increase in dihydroxyacetone phosphate production was selected from a library of random mutations (Hu et al. 2021). Another directed evolution study found increased activity by 4.7-fold and a preference for DHA at low formaldehyde concentrations (5 mM) (Cai et al. 2021). Li et al. screened approximately 8,000 transformants and isolated a variant with a 9.2-fold improvement in catalytic efficiency (*k*_cat_/*K*_m_) (Li et al. 2020).

In this study, instead of directed evolution, scanning saturation mutagenesis was used to investigate the sequence-function correlation and identify key residues essential for the C1 to C3 conversion by FLS derived from *P. putida* BFD (Li et al. 2020; Lu et al. 2019). Twenty-five residues within an 8 Å distance from the active center were screened via single-point saturation mutagenesis. Notably, this method enabled the identification of several beneficial sites, some of which were not identified in previous directed evolution studies. Molecular dynamics (MD) simulations were conducted to analyze the effects of amino acid substitutions on the enzyme structure and catalytic activity. This information will provide guidance for the future engineering of BFD-derived FLS to enhance the activity.

## Materials and methods

### Bacterial strains, plasmids, and growth conditions

*Escherichia coli* strains Trans1-T1 and BL21 (DE3) (TransGen Biotech, Beijing, China) were used for plasmid cloning and protein expression, respectively. *E. coli* was cultivated aerobically at 37ºC in Luria Bertani (LB) medium, which was supplemented with kanamycin (50 µg/mL) and 0.1 mM isopropyl β-d‐thiogalactoside as required. The pET28a-*GALS* plasmid containing the original FLS (*P. putida* BFD with W86R/N87T/L109G/L110E/A460M mutations) (Lu et al. 2019) was used as a template to construct the FLS variants. The nucleotide and amino acid sequences of the original FLS are listed in Table S1.

### Single-site saturation mutagenesis and variant screening

Single-site saturation mutagenesis was conducted according to the PCR-based Quick Change method, following a previously described procedure using the pET28a-*GALS* plasmid as the template (Lu et al. 2019). Oligonucleotide primers were designed using the degenerate codon, NNK (N = A/T/C/G, K = G/T). Approximately 200 transformants were screened for each single-site saturation mutagenesis library. The transformants were randomly picked and cultivated in 200 µL LB medium (containing 50 µg/mL kanamycin) in 96-deep-well microplates. After cultivation at 37ºC and 800 rpm for 24 h, the cultures were used as seed cultures to inoculate 200 µL fresh LB medium (containing 50 µg/mL kanamycin and 0.1 mM IPTG) in 96-deep-well microplates. Subsequently, the cells were cultured at 37ºC and 800 rpm for 4 h, and the temperature was reduced to 30ºC for another 24 h of cultivation. Then, the pellets were harvested by centrifugation (3,400 rpm for 15 min) and then resuspended in phosphate buffer (100 mM HEPES-NaCl, pH 7.4, 50 µL). For activity screening, 50 µL formaldehyde solution containing 1 mM thiamine diphosphate (TPP) was added to the above plates. After incubation at 30ºC for 3 h, the plates were centrifuged (3,400 rpm for 15 min) and 20 µL supernatant was transferred into 96-well microtiter plates containing 80 µL reaction mix for DHA measurement. The reaction mix contained 50 mM Tris-HCl (pH 7.0), 10 µM flavin adenine dinucleotide, 0.05 mg/mL horseradish peroxidase, 0.2 mg/mL 2,4,6-tribromo-3-hydroxybenzoic acid, 0.15 mg/mL 4-aminoantipyrine, and 0.0325 mg/mL galactose oxidase. Following incubation at 30ºC for 1 h, the OD_510nm_ was measured (Fig. S1). This method was demonstrated to specifically quantify DHA levels without interference from GA (Fig. S2). Mutants exhibiting relatively higher OD_510nm_ values were considered to exhibit high DHA production rates and thus selected for subsequent analyses.

### FLS expression, purification, and activity assay

*E. coli* BL21 (DE3) strains harboring the recombinant plasmids were grown in 50 mL LB medium (containing 50 µg/mL kanamycin) in 250 mL Erlenmeyer flasks at 37ºC and 220 rpm. When the OD_600_ of the culture reached 1.0, 0.1 mM IPTG was added to induce gene expression. Subsequently, the cultivation temperature was changed to 16ºC. After 20 h of cultivation, the cells were harvested by centrifugation at 6,000 rpm for 15 min and resuspended in lysis buffer (50 mM KH_2_PO_4_-K_2_HPO_4_, 5 mM MgSO_4_, pH 7.8). Cells were lysed by ultrasonication, and the cell lysate was centrifuged at 17,000 rpm for 45 min. The FLS protein in the supernatants was purified using His-Spin protein mini-prep columns (Zymo Research, USA) and manipulated in lysis buffer. The protein concentration was determined using a BCA protein assay kit (Pierce, USA) with bovine serum albumin as the standard. The kinetic constants of FLS were determined by coupling glycerol dehydrogenase to reduce the production of DHA and GA while concurrently consuming NADH (Lu et al. 2019). Because the glycerol dehydrogenase employed in the kinetic assay can react with both DHA and GA, the ratio between DHA and GA was further determined using high-performance liquid chromatography (HPLC), according to a previously described procedure (Cai et al. 2021).

### Structural modeling and MD simulations

RosettaLigand-based mutant structural modeling was conducted following a previously described procedure (Lu et al. 2019). Figures depicting enzyme structures and interactions were visualized using the PyMOL software (PyMOL Molecular Graphics System, Schrödinger, LLC). The mutant complex structures were set as the initial structures for MD simulations. Protein structures were prepared using the pdb4amber application in the Amber20 package (Case et al. 2021). The force field for the intermediate analog was generated by Antechamber using the AM1-BCC charge model (Jakalian et al. 2002; Wang et al. 2001). A small amount of Na^+^ ions were introduced onto the protein surface to neutralize the overall charge of the system. Ultimately, the resulting system was solvated in a rectangular box filled with TIP3P water, ensuring a minimum cutoff distance of 8 Å from the protein boundary. For all proteins, the Amber ff14SB force field was employed throughout the MD simulations.

After proper parameterization and setup, the resultant systems underwent minimization in two steps to eliminate the weak contacts and relax the systems: the first step consisted of 5,000 steps of steepest descent and 10,000 steps of conjugate gradient, while the second step consisted of 10,000 steps of steepest descent and 30,000 steps of conjugate gradient. Next, the systems were gradually annealed from 0 to 300 K under the NVT ensemble for 50 ps, with a restraint of 5.0 kcal/(mol·Å). Subsequently, the systems were maintained in the NPT ensemble for a density equilibration of 20 ps at a target temperature of 300 K and a target pressure of 1.0 atm using the Langevin thermostat (Larini et al. 2007) with a restraint of 1.0 kcal/(mol·Å). Thereafter, all of the restraints applied during heating and density dynamics were removed, and the system underwent further equilibration for ∼2 ns to ensure stable pressure and temperature for subsequent conformational and chemical analyses. Each system then underwent a 200 ns MD production run. Throughout all MD simulations, covalent bonds containing hydrogen were constrained using SHAKE (Ryckaert et al. 1977), and particle-mesh Ewald (Darden and York 1993) was employed to stimulate long-range electrostatic interactions. All the MD simulations were performed using the GPU version of the Amber 20 package.

## Results and discussion

### Screening of FLS variants with improved DHA formation from single-site saturation mutation libraries

The BFD from *P. putida* was modified to perform the carboligation of multiple formaldehyde molecules, with C2 GA as the major product and DHA as the minor product (Lu et al. 2019). To identify key residuals required for enhancing DHA formation activity, 25 residues within an 8 Å distance from the active center that may have an impact on the enzyme activity were selected, and single-point saturation mutagenesis was conducted (Fig. 1B). To cover all possible amino acid substitutions, approximately 200 transformants expressing recombinant FLS variants were selected and tested for DHA production from formaldehyde. A color reaction for DHA quantification without GA interference was developed to increase the throughput of variant screening (Figs. S1 and S2). Transformants producing higher DHA than the control strain harboring the original FLS were further subjected to PCR amplification and DNA sequencing (Fig. S3A). By screening ∼ 5,000 transformants, we identified 10 amino acid substitutions at six sites (S26F, G109I, S236F, S236L, H281Y, C398M, C398A, G401L, G401H, and G401Y), which improved the DHA formation activity of FLS by 1.5- to 5.3-fold in whole-cell biocatalysis (Fig. S3B). Given that no biological replicates were conducted for the initial round of screening of ∼ 5,000 transformants, the seven FLS variants demonstrating over a 2-fold improvement in DHA formation (S26F, G109I, S236F, S236L, H281Y, C398M, and C398A) were subjected to re-testing for whole-cell biocatalysis, with three biological replicates conducted. These seven FLS variants exhibited higher activity to the original enzyme in terms of DHA formation, although the levels of improvement were not exactly the same as those observed during the initial round of screening (Fig. 1C). These variations are typical when screening large numbers of different enzyme variants; thus, multiple rounds of screening are typically required in such cases (Qian et al. 2023; Yang et al. 2024). Interestingly, these selected sites partially overlapped with previous findings through directed evolution. FLS variants harboring the S26F/H281Y and S26F/G109S/H281Y mutations were selected from random mutation libraries to increase DHA formation activity (Li et al. 2020). Moreover, several new beneficial sites and amino acid substitutions were identified, including G109I, S236F, S236L, C398M, and C398A, which were not previously identified by directed evolution.

Directed evolution is a powerful strategy for obtaining desired enzyme variants based on random mutation and activity screening (Chen et al. 2022; Guan et al. 2024). However, considering that the theoretical library size of randomly mutated FLS is extremely large (528^20^=2.8 × 10^54^), it is impractical to cover every possible mutation during library construction and screening. Consequently, it is possible that residue substitutions that would improve the catalytic activity may go undetected during the initial screening process. In this study, the active center of FLS was scanned using single-point saturation mutagenesis to identify the key residues for C1 to C3 bioconversion activity. Some beneficial residue substitutions that were not covered during previous directed evolution studies were identified, suggesting the complementation of these two enzyme engineering strategies.

### Kinetic characterization of selected FLS variants

Seven FLS variants exhibiting over a 2-fold improvement in DHA formation (S26F, G109I, S236F, S236L, H281Y, C398M, and C398A) were overexpressed and purified to characterize their kinetic constants (Fig. S4). Except for the C398A variant, the other variants displayed 1.4- to 2.4-fold increases in affinity for formaldehyde (Table 1 and Fig. S5). These results are consistent with those of a previous directed evolution study in which a combination of H281Y/S26F mutations resulted in a 1.7-fold improvement in formaldehyde affinity. (Li et al. 2020). High-affinity variants are frequently obtained because of the formaldehyde diffusion limitations of the cell membrane. Therefore, variants exhibiting higher affinity for formaldehyde are more conducive to binding and catalyzing the intracellular formaldehyde required for carboligation. Considering that formaldehyde is a highly active and cytotoxic compound, the accumulation of high concentrations of formaldehyde is lethal to microbial cells (Zhang et al. 2021). In addition, the oxidation of formaldehyde to formate and the reduction of formaldehyde to methanol are both more thermodynamically feasible compared to formaldehyde-generating reactions (Wang et al. 2020). Consequently, the accumulation of formaldehyde at high levels is challenging, even in in vitro catalytic systems. Therefore, increasing the substrate affinity of FLS is crucial for its application in C1 bioconversion.

**Table 1:** Apparent kinetic constants of purified FLS variants

AA mutation	*k*_cat_ (min^− 1^)^b^	*K*_M_ (mM)^b^	*k*_cat_/*K*_M_ (min^− 1^·mM^− 1^)^b^
WT^a^	73.1 ± 0.1	88.7 ± 3.1	0.82 ± 0.03
S26F	57.2 ± 0.2	62.1 ± 1.3	0.92 ± 0.02
G109I	55.0 ± 0.9	49.6 ± 0.7	1.11 ± 0.01
S236F	64.1 ± 2.7	56.9 ± 5.1	1.12 ± 0.05
S236L	50.5 ± 1.0	37.7 ± 2.9	1.34 ± 0.08
H281Y	69.1 ± 0.9	61.6 ± 4.3	1.12 ± 0.06
C398A	79.0 ± 0.7	90.7 ± 2.8	0.87 ± 0.02
C398M	74.1 ± 2.2	62.3 ± 3.7	1.19 ± 0.04

^a^WT represents the original FLS (*P. putida* BFD with W86R/N87T/L109G/L110E/A460M mutations)

^b^Values and error bars reflect the mean ± s.d. of three biological replicates

### Determination of the product preference of selected FLS variants

**Fig. 2:**
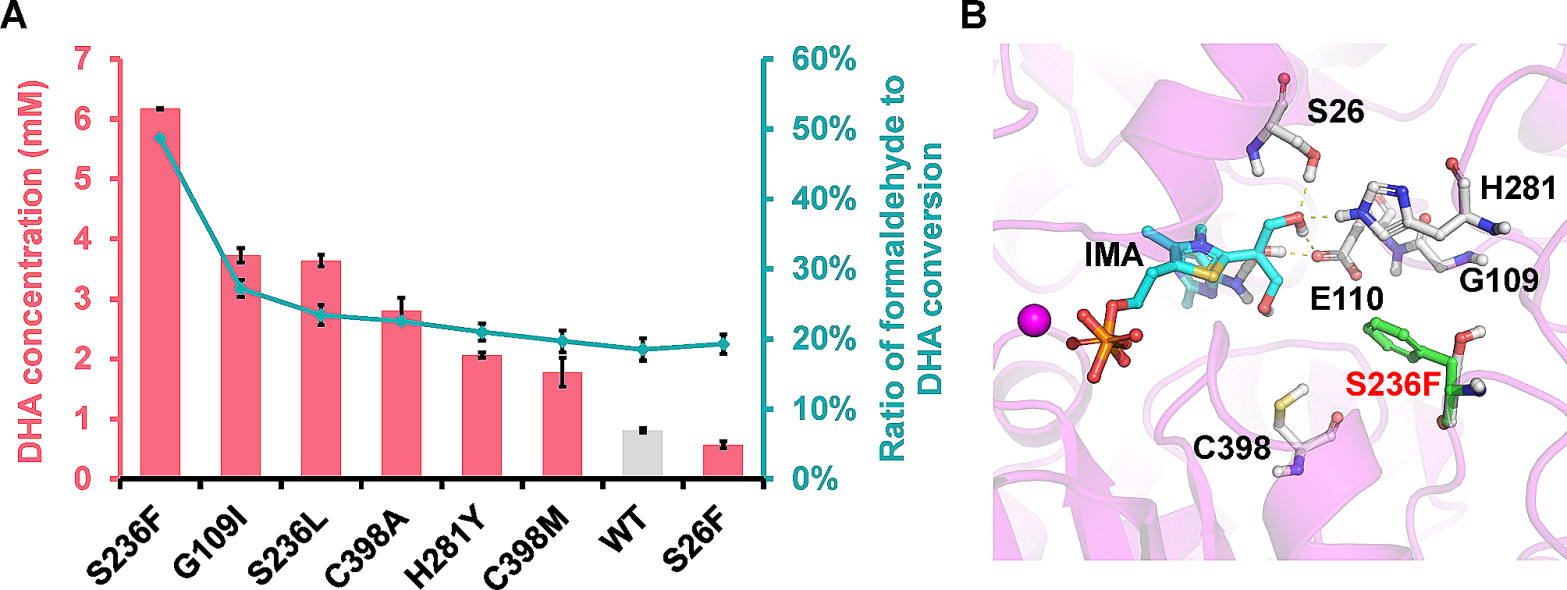
Characterization of the selected FLS variants. **A** Product preference of selected FLS variants. 75 mM formaldehyde was used as the substrate. Values and error bars reflect the mean ± s.d. of three biological replicates. **B** Analysis of the effects of the S236F mutation on structure using MD simulations. The intermediate analog (IMA) is accentuated in cyan, while the S236F mutation is represented in green using a stick-and-ball model. Hydrogen bonds between the hydroxyl group of IMA and the residues E110, S26, and H281 are illustrated with yellow dashes

To determine the initial velocity of formaldehyde carboligation, glycerol dehydrogenase was used, which catalyzes the reduction of both GA and DHA (Lu et al. 2019). The *k*_cat_ values determined cannot accurately reflect the DHA formation activity. Therefore, to determine the DHA formation activity of the selected variants and their DHA/GA preference, the products of the enzymatic reactions were subjected to HPLC analysis. With the exception of S26F, the remaining six FLS variants produced higher concentrations of DHA compared to the wild-type FLS (Fig. 2A and Fig. S6). The FLS^S236F^ variant outperformed the other variants and wild-type FLS in terms of both DHA formation activity and DHA preference against GA, which were 7.6- and 2.6-fold higher than those of the wild-type FLS, respectively. However, the kinetic constants of FLS^S236F^ were not significantly higher than those of the original enzyme (Table 1). The inconsistency between these two assays is attributed to the fact that the glycerol dehydrogenase used to determine the kinetic constants can react with both GA and DHA as substrates (Lu et al. 2019). Consequently, HPLC analysis of the reaction products was required to accurately determine DHA production. Additionally, we observed discrepancies between the DHA formation activities determined via whole-cell biocatalysis and those determined by the purified enzyme reaction, which could be potentially attributed to the variations in the expression levels of FLS variants or the different conditions between in vivo and in vitro reactions. Although a previous directed evolution study has suggested that S26F mutations improve DHA production when combined with H281Y and G109S/H281Y (Li et al. 2020), our current research indicates that individual S26F mutations may not be as effective (Fig. S7).

To investigate the mechanism underlying the improved DHA formation activity and preference for DHA, RosettaLigand-based mutant structural modeling was conducted based on the crystal structure of the parent FLS (Lu et al. 2019) and MD simulations were performed. The S236F mutation notably increased local hydrophobicity at the active site, predisposing the intermediate analog to nucleophilic attack by the third formaldehyde molecule (Fig. 2B). This could facilitate subsequent DHA generation with the third formaldehyde molecule. MD simulations of the other mutants revealed varying degrees of increased hydrophobicity in the binding pocket. In addition, the H281Y mutation concurrently increased the probability of forming a hydrogen bond with an intermediate analog at Y281. This alteration not only stabilizes the intermediate analog, but also enhances its susceptibility to nucleophilic attack by the third formaldehyde molecule (Fig. S8). Therefore, reshaping the hydrophobicity of the active site plays a crucial role in improving C1 to C3 bioconversion by FLS.

## Conclusions

In this study, 25 residues adjacent to the active center of FLS were systematically screened to investigate their effects on DHA formation activity. New sites and amino acid substitutions were identified for their positive effects on DHA formation and formaldehyde affinity. The identified mutations can be combined either with each other or with mutations obtained from previous directed evolution studies to test their combined effects on enzyme activity. The top performing FLS^S236F^ variant produced 7.4-fold higher DHA from formaldehyde compared to the wild-type FLS. This study provided a mechanism to provide guidance for further engineering of this crucial enzyme performing the C1-bioconversion process.

### Electronic supplementary material

Below is the link to the electronic supplementary material.

Supplementary Material 1

Supplementary Material 2

## Data Availability

The datasets supporting the conclusions of this article are included within the article and its additional files.

## Abbreviations

DHA: Dihydroxyacetone
FLS: Formolase
GA: Glycolaldehyde
HPLC: High performance liquid chromatography
MD: Molecular dynamics
TPP: Thiamine diphosphate
