# Supplementary material for: Scanning the active center of formolase to identify key residues for enhanced C1 to C3 bioconversion
Source: Bioresour Bioprocess. 2024 May 12;11(1):48. doi: 10.1186/s40643-024-00767-3 (PMC11089019; doi:10.1186/s40643-024-00767-3)
Supplement: Supplementary file 1 — Supplementary Material 1 [file 40643_2024_767_MOESM1_ESM.docx]

**Additional file 1 for**

**Scanning the active center of formolase to identify key residues for enhanced C1 to C3 bioconversion**

**
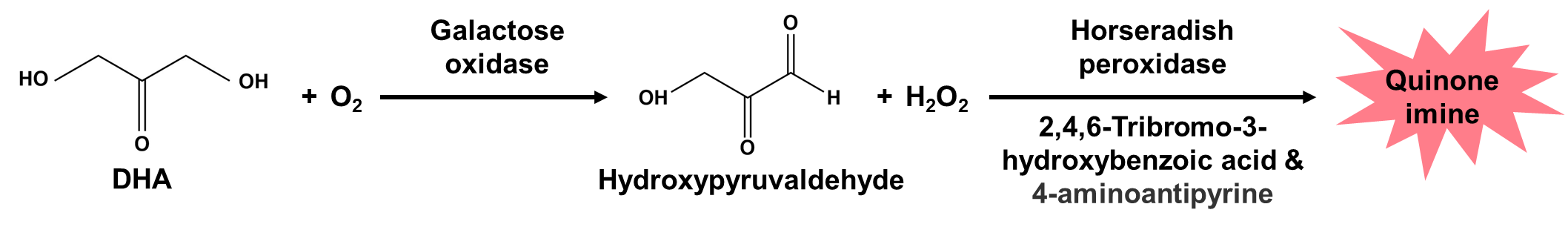
**

**Fig. S1.** The color reaction used for DHA quantification.

**
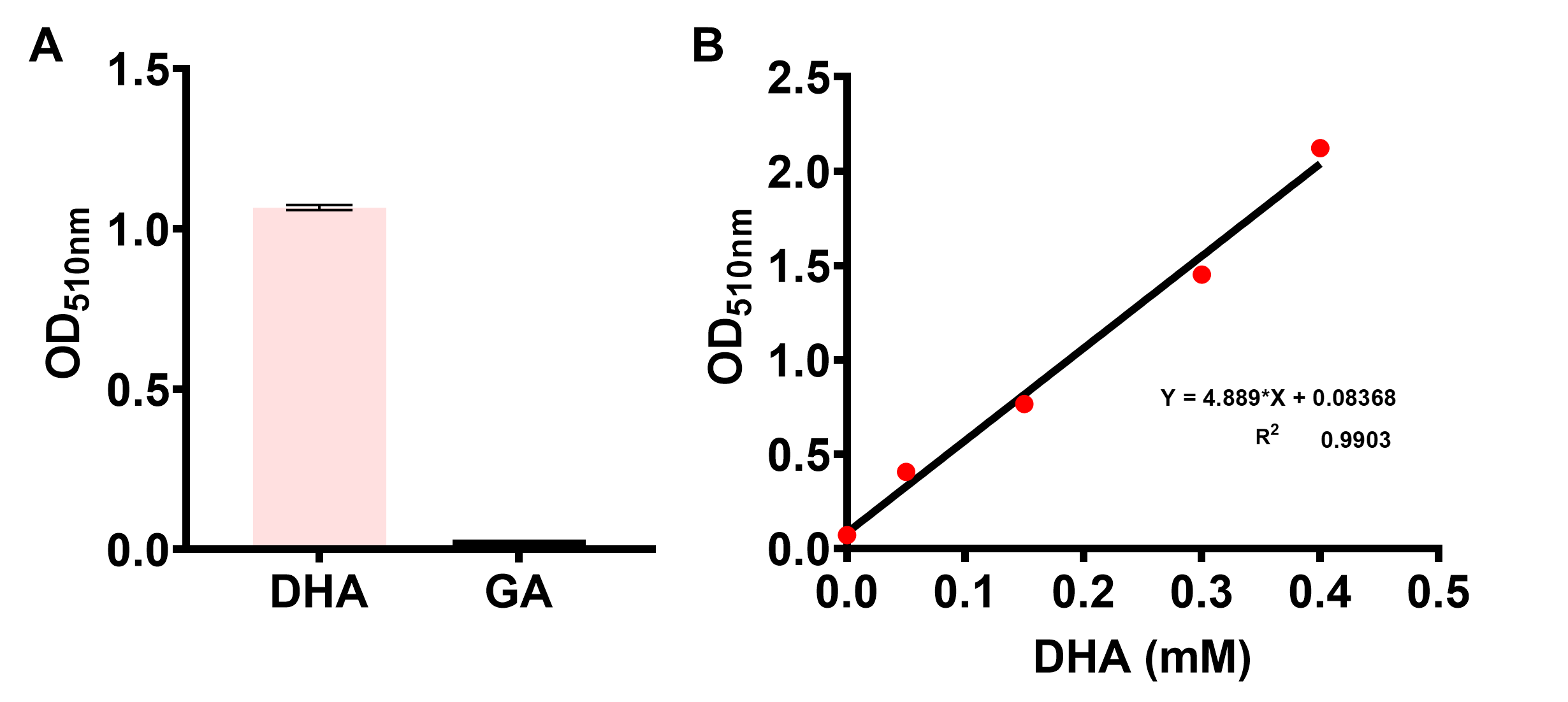
**

**Fig. S2.** Quantification of DHA by using the color reaction described in the Materials and Methods section. **A** Specificity of DHA measurement. 0.2 mM DHA or GA was determined using the color reaction. Values and error bars reflect the mean ± s.d. of three biological replicates. **B** Standard curve for DHA. The reaction mix contains 50 mM Tris-HCl (pH 7.0), 10 μM flavin adenine dinucleotide, 0.05 mg/mL horseradish peroxidase, 0.2 mg/mL 2,4,6-tribromo-3-hydroxybenzoic acid, 0.15 mg/mL 4-aminoantipyrine, 0.0325 mg/mL galactose oxidase. After incubation at 30ºC for 1 hour, OD_510nm_ was determined. Values and error bars reflect the mean ± s.d. of three biological replicates.


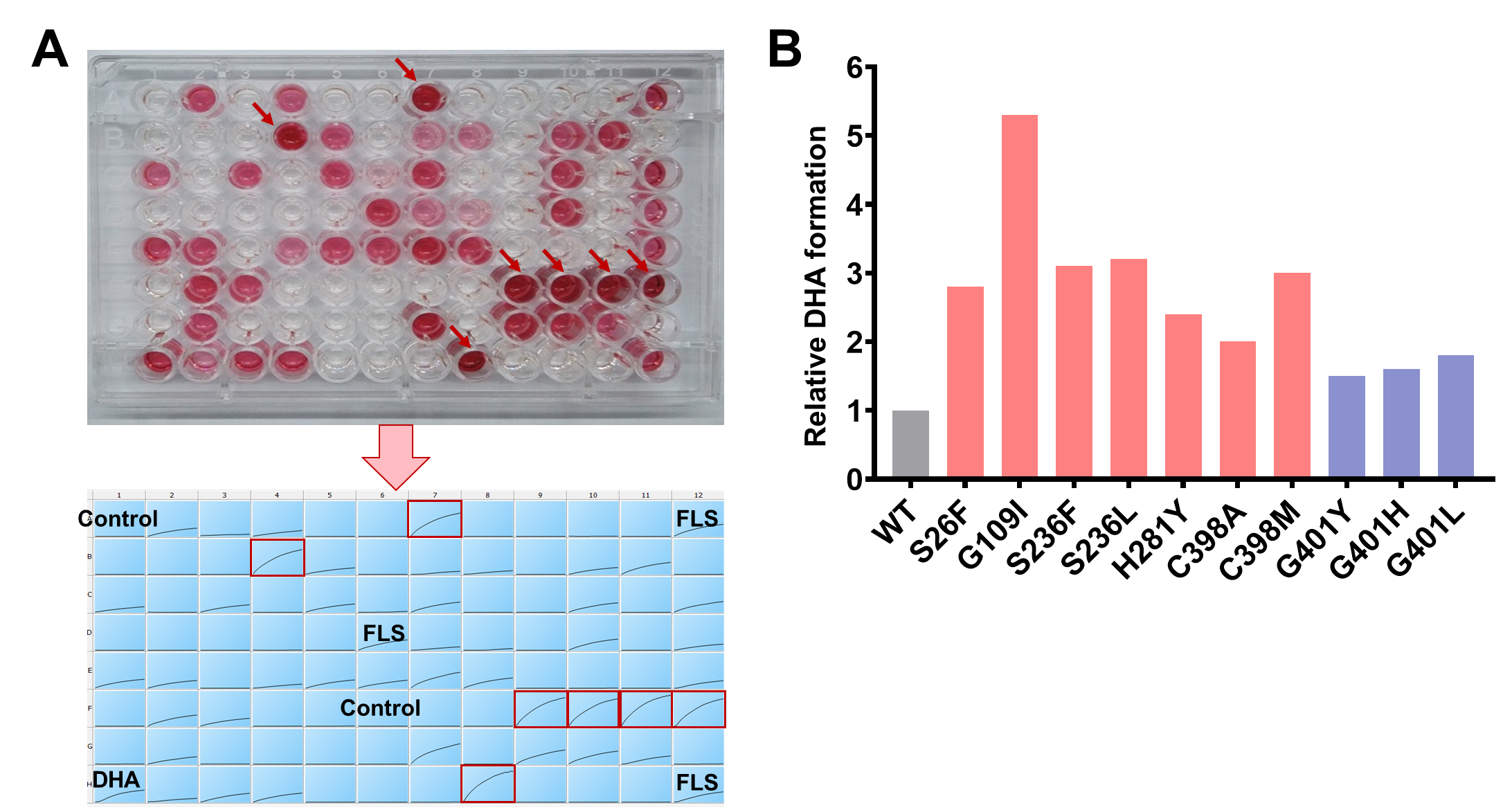


**Fig. S3.** Screening of FLS variants with improved DHA formation. **A** A representative 96-well plate for screening FLS variants using the color reaction for DHA quantification was shown. DHA formation was quantified by measuring OD_510nm_ using a microplate reader after adding the reaction mix. Control and FLS represent *E. coli* strains harboring an empty plasmid and an original FLS-expressing plasmid, respectively. DHA represents positive control supplemented with 0.3 mM DHA. **B** Ten amino acid substitutions beneficial for DHA formation were identified by the first round of screening of FLS variants. The seven FLS variants with over 2-fold improvement in DHA formation were marked in red and subjected for the second round of screening.

**
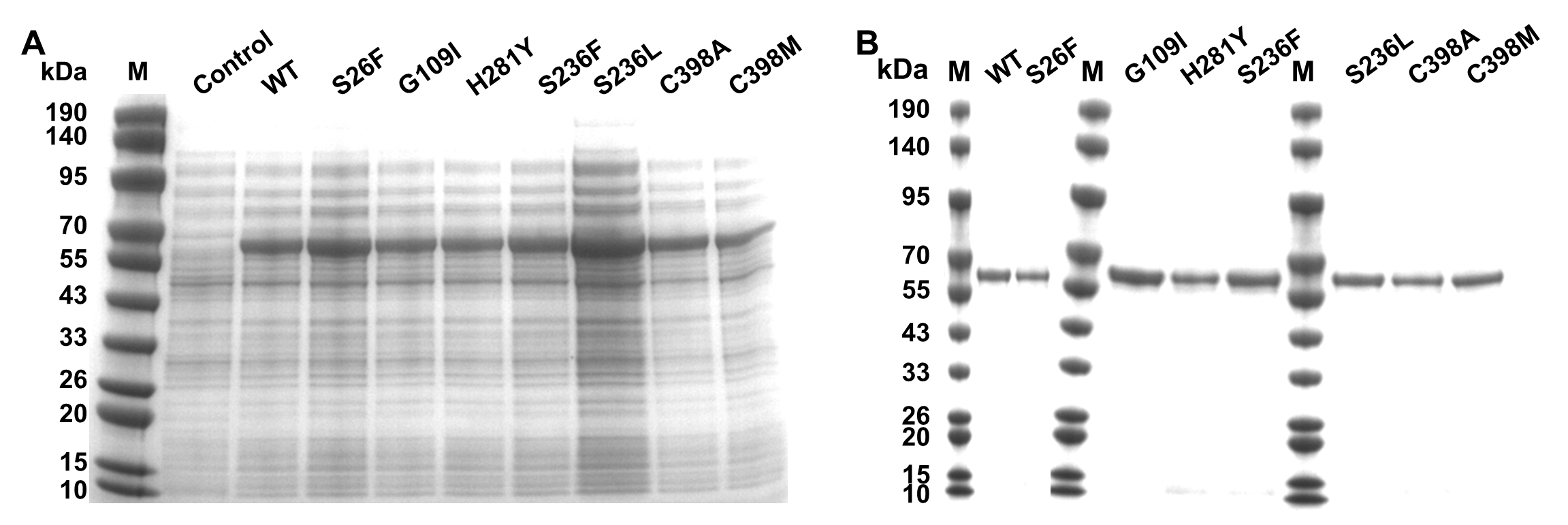
**

**Fig. S4.** SDS-PAGE analysis of FLS expression and purification. **A** SDS-PAGE analysis of supernatants of cell lysates. **B** SDS-PAGE analysis of purified FLS enzymes.

**
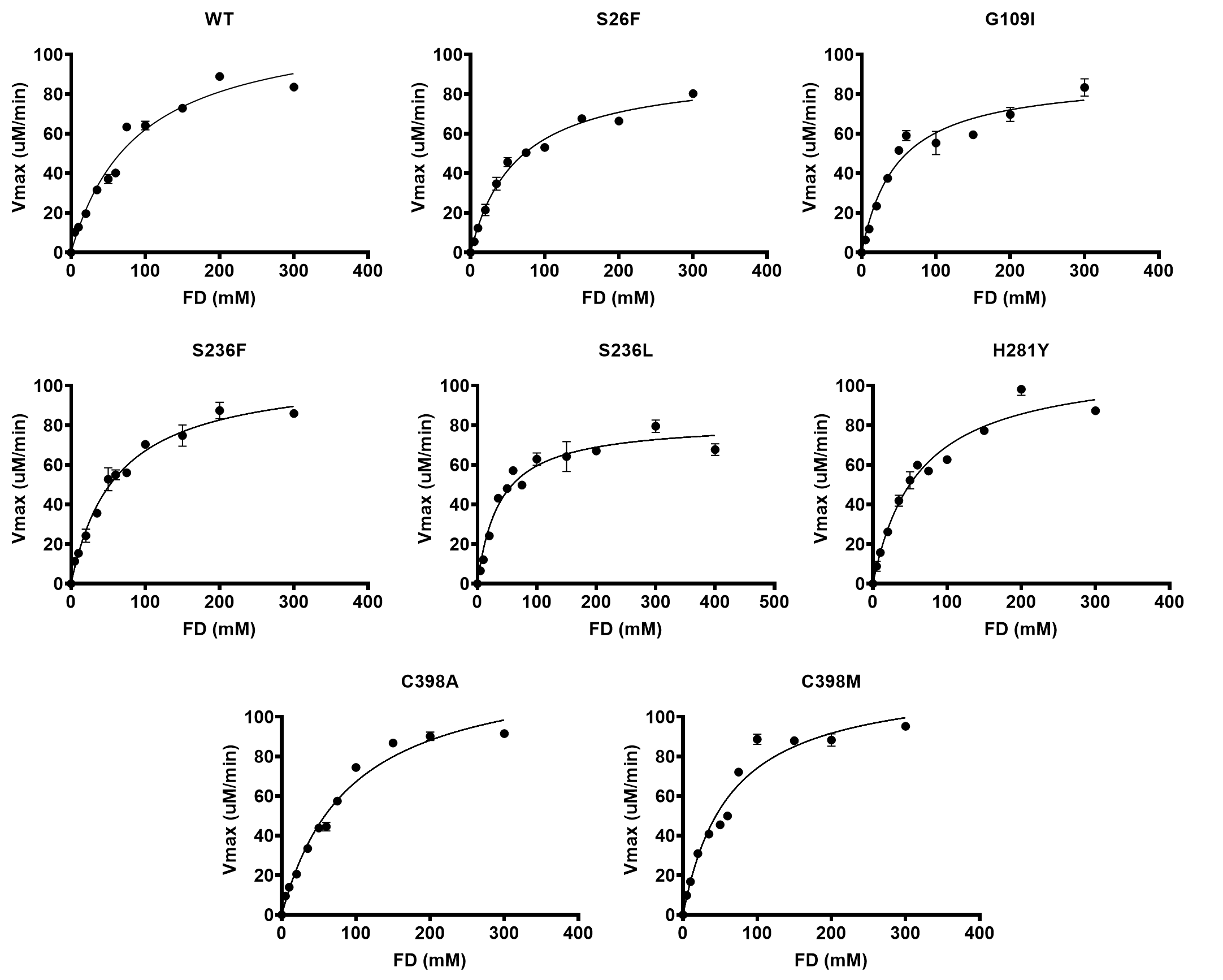
**

**Fig. S5.** Kinetic assay of the original FLS and variants. Values and error bars reflect the mean ± s.d. of three biological replicates.

**
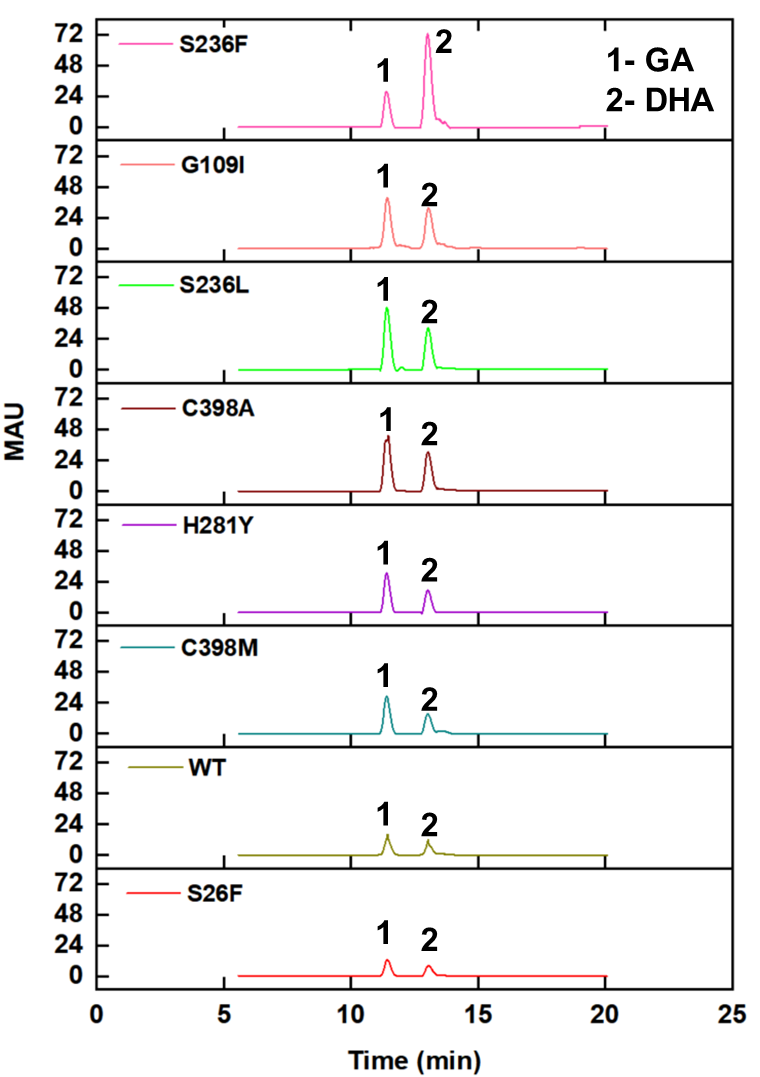
**

**Fig. S6.** Determination of DHA and GA produced by purified FLS variants by HPLC.


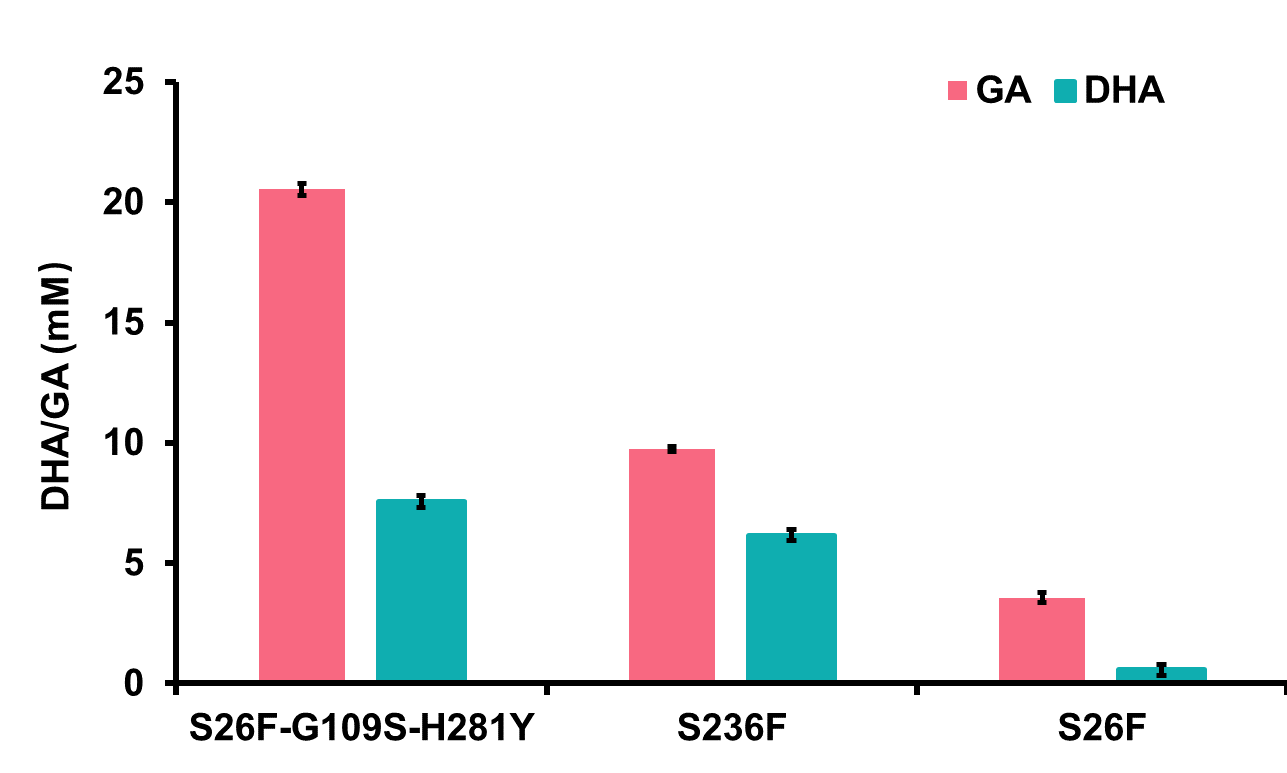


**Fig. S7.** Comparison of FLS variants obtained by directed evolution and single-point saturation mutagenesis. 75 mM formaldehyde was used as the substrate. Values and error bars reflect the mean ± s.d. of three biological replicates.


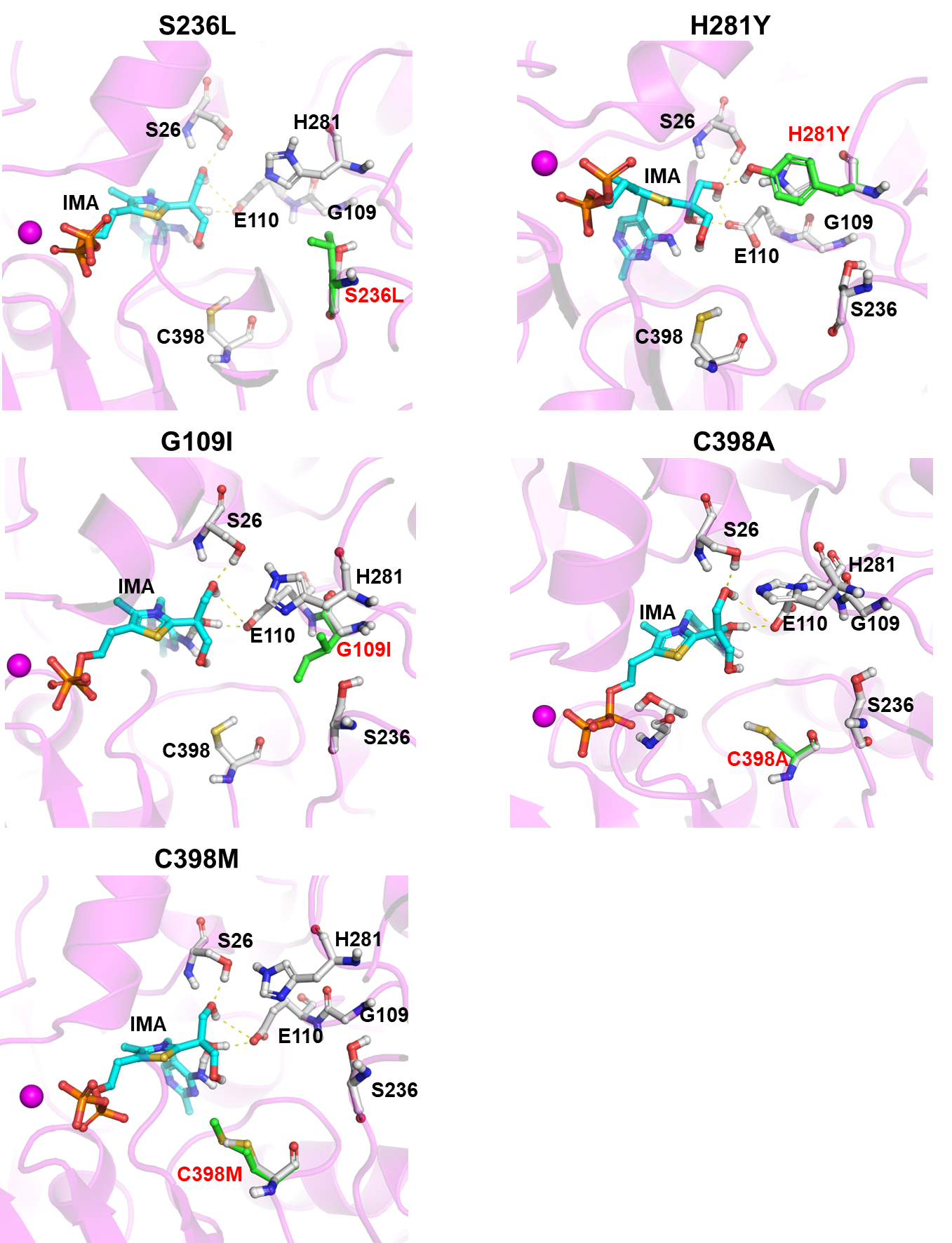


**Fig. S8.** Analysis of the effects of amino acid substitutions on structure by MD simulations. The intermediate analogue (IMA) is accentuated in cyan, while the mutation is represented in green using a stick-and-ball model. Hydrogen bonds between the hydroxyl group of IMA and the residues E110, S26, and H281 (or Y281) are illustrated with yellow dashes.

**Table S1.** The nucleotide and amino acid sequence of the wild-type FLS.

| **DNA sequence** | ATGGCTTCTGTTCACGGTACCACCTACGAACTGCTGCGTCGTCAGGGTATCGACACCGTTTTCGGTAACCCGGGTTCTAACGAACTGCCGTTCCTGAAAGACTTCCCGGAAGACTTCCGTTACATCCTGGCTCTGCAGGAAGCTTGCGTTGTTGGTATCGCTGACGGTTACGCTCAGGCTTCTCGTAAACCGGCTTTCATCAACCTGCACTCTGCTGCTGGTACCGGTAACGCTATGGGTGCTCTGTCTAACGCTCGTACCTCTCACTCTCCGCTGATCGTTACCGCTGGTCAGCAGACCCGTGCTATGATCGGTGTTGAAGCTGGTGAAACCAACGTTGACGCTGCTAACCTGCCGCGTCCGCTGGTTAAATGGTCTTACGAACCGGCTTCTGCTGCTGAAGTTCCGCACGCTATGTCTCGTGCTATCCACATGGCTTCTATGGCTCCGCAGGGTCCGGTTTACCTGTCTGTTCCGTACGACGACTGGGACAAAGACGCTGACCCGCAGTCTCACCACCTGTTCGACCGTCACGTTTCTTCTTCTGTTCGTCTGAACGACCAGGACCTGGACATCCTGGTTAAAGCTCTGAACTCTGCTTCTAACCCGGCTATCGTTCTGGGTCCGGACGTTGACGCTGCTAACGCTAACGCTGACTGCGTTATGCTGGCTGAACGTCTGAAAGCTCCGGTTTGGGTTGCTCCGTCTGCTCCGCGTTGCCCGTTCCCGACCCGTCACCCGTGCTTCCGTGGTCTGATGCCGGCTGGTATCGCTGCTATCTCTCAGCTGCTGGAAGGTCACGACGTTGTTCTGGTTATCGGTGCTCCGGTTTTCCGTTACCACCAGTACGACCCGGGTCAGTACCTGAAACCGGGTACCCGTCTGATCTCTGTTACCTGCGACCCGCTGGAAGCTGCTCGTGCTCCGATGGGTGACGCTATCGTTGCTGACATCGGTGCTATGGCTTCTGCTCTGGCTAACCTGGTTGAAGAATCTTCTCGTCAGCTGCCGACCGCTGCTCCGGAACCGGCTAAAGTTGACCAGGACGCTGGTCGTCTGCACCCGGAAACCGTTTTCGACACCCTGAACGACATGGCTCCGGAAAACGCTATCTACCTGAACGAATCTACCTCTACCACCGCTCAGATGTGGCAGCGTCTGAACATGCGTAACCCGGGTTCTTACTACTTCTGCGCTGCTGGTGGTCTGGGTTTCGCTCTGCCGGCTGCTATCGGTGTTCAGCTGGCTGAACCGGAACGTCAGGTTATCGCTGTTATCGGTGACGGTTCTGCTAACTACTCTATCTCTGCTCTGTGGACCGCTGCTCAGTACAACATCCCGACCATCTTCGTTATCATGAACAACGGTACCTACGGTATGCTGCGTTGGTTCGCTGGTGTTCTGGAAGCTGAAAACGTTCCGGGTCTGGACGTTCCGGGTATCGACTTCCGTGCTCTGGCTAAAGGTTACGGTGTTCAGGCTCTGAAAGCTGACAACCTGGAACAGCTGAAAGGTTCTCTGCAGGAAGCTCTGTCTGCTAAAGGTCCGGTTCTGATCGAAGTTTCTACCGTTTCTCCGGTTAAATAA |
| --- | --- |
| **AA sequence** | MASVHGTTYELLRRQGIDTVFGNPGSNELPFLKDFPEDFRYILALQEACVVGIADGYAQASRKPAFINLHSAAGTGNAMGALSNARTSHSPLIVTAGQQTRAMIGVEAGETNVDAANLPRPLVKWSYEPASAAEVPHAMSRAIHMASMAPQGPVYLSVPYDDWDKDADPQSHHLFDRHVSSSVRLNDQDLDILVKALNSASNPAIVLGPDVDAANANADCVMLAERLKAPVWVAPSAPRCPFPTRHPCFRGLMPAGIAAISQLLEGHDVVLVIGAPVFRYHQYDPGQYLKPGTRLISVTCDPLEAARAPMGDAIVADIGAMASALANLVEESSRQLPTAAPEPAKVDQDAGRLHPETVFDTLNDMAPENAIYLNESTSTTAQMWQRLNMRNPGSYYFCAAGGLGFALPAAIGVQLAEPERQVIAVIGDGSANYSISALWTAAQYNIPTIFVIMNNGTYGMLRWFAGVLEAENVPGLDVPGIDFRALAKGYGVQALKADNLEQLKGSLQEALSAKGPVLIEVSTVSPVK* |
