# Supplementary figures and images for: Scanning the active center of formolase to identify key residues for enhanced C1 to C3 bioconversion
Source: Bioresour Bioprocess. 2024 May 12;11(1):48. doi: 10.1186/s40643-024-00767-3 (PMC11089019; doi:10.1186/s40643-024-00767-3)

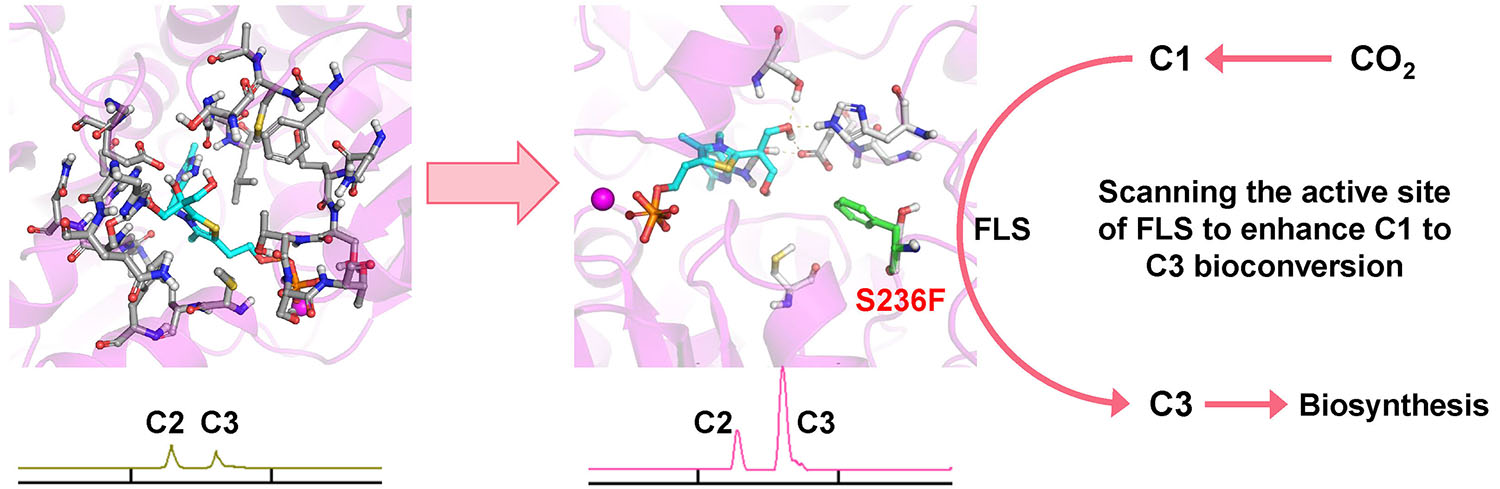

Supplement: Supplementary file 2 — Supplementary Material 2 [file 40643_2024_767_MOESM2_ESM.jpg]
